# Population structure and genetic history of Tibetan Terriers

**DOI:** 10.1186/s12711-019-0520-4

**Published:** 2019-12-27

**Authors:** Mateja Janeš, Minja Zorc, Vlatka Cubric-Curik, Ino Curik, Peter Dovc

**Affiliations:** 1 Department of Animal Science, University of Zagreb, Faculty of Agriculture, Zagreb, Croatia; 2 Department of Animal Science, University of Ljubljana, Biotechnical Faculty, Ljubljana, Slovenia

## Abstract

**Background:**

Tibetan Terrier is a popular medium-sized companion dog breed. According to the history of the breed, the western population of Tibetan Terriers includes two lineages, Lamleh and Luneville. These two lineages derive from a small number of founder animals from the native Tibetan Terrier population, which were brought to Europe in the 1920s. For almost a century, the western population of Tibetan Terriers and the native population in Tibet were reproductively isolated. In this study, we analysed the structure of the western population of Tibetan Terriers, the original native population from Tibet and of different crosses between these two populations. We also examined the genetic relationships of Tibetan Terriers with other dog breeds, especially terriers and some Asian breeds, and the within-breed structure of both Tibetan Terrier populations.

**Results:**

Our analyses were based on high-density single nucleotide polymorphism (SNP) array (Illumina HD Canine 170 K) and microsatellite (18 loci) genotypes of 64 Tibetan Terriers belonging to different populations and lineages. For the comparative analysis, we used 348 publicly available SNP array genotypes of dogs from other breeds. We found that the western population of Tibetan Terriers and the native Tibetan Terriers clustered together with other Asian dog breeds, whereas all other terrier breeds were grouped into a separate group. We were also able to differentiate the western Tibetan Terrier lineages (Lamleh and Luneville) from the native Tibetan Terrier population.

**Conclusions:**

Our results reveal the relationships between the western and native populations of Tibetan Terriers and support the hypothesis that Tibetan Terrier belongs to the group of ancient dog breeds of Asian origin, which are close to the ancestors of the modern dog that were involved in the early domestication process. Thus, we were able to reject the initial hypothesis that Tibetan Terriers belong to the group of terrier breeds. The existence of this native population of Tibetan Terriers at its original location represents an exceptional and valuable genetic resource.

## Background

Tibetan Terrier (TT) is a medium-sized companion dog breed, which is present in many countries all over the world. The so-called western population of Tibetan Terriers originated from a small number of founder animals that were imported from the border region between Tibet and India (Central Himalaya) at the beginning of the twentieth century [1]. In western countries, two lineages were established during the history of the breed, the older Lamleh lineage, which can be traced back to the first two animals (Bunti and Rajah) that were acquired by Dr. Agnes Greig in 1922 and brought to England in 1930. In 1937, the Kennel Club of England recognized the Tibetan Terrier as an own breed, based on animals belonging to the Lamleh lineage. The second lineage of Tibetan Terriers i.e. Luneville was formed by mating Dusky, a stray dog, found by John Downey in Liverpool in 1953 (it was registered as a Tibetan Terrier and named Trojan Kynos) and the bitch Princess Aureus [2] (see Additional file 1: Figure S1). Both lineages go back to a very limited number of founders from the native Tibetan Terrier (TTNA) population and represent the link between the western population of Tibetan Terriers and the original population in Tibet, which supports the genetic relationship between both populations. However, following recognition of the western population of Tibetan Terriers by kennel clubs in Europe, the United States, Australia and other countries, their population underwent a long and relative strict isolation from the original native population of Tibetan Terriers in Tibet. This isolation was broken only sporadically through a few imports of single animals, which refreshed temporarily the western population. Consequently, a very special situation was created for the western population of Tibetan Terriers, which is a pedigree-controlled population that exists in parallel to the original native population in Tibet. It is only very recently that a reproductive contact between both populations occurred through a limited number of imported dogs from Tibet to the western countries. The western population of Tibetan Terriers is registered at The International Cynological Organisation [Fédération Cynologique Internationale (FCI), Breed standard N° 209, 2017]. The original native population of Tibetan Terriers is still present in Tibet and represents the original gene pool from which only a few individuals contributed as founders to the western population of Tibetan Terriers. In addition, the native population of Tibetan Terriers contributed also to the formation of the gene pool of several other Tibetan dog breeds, i.e. Lhasa Apso, Shih Tzu, and Tibetan Spaniel, which are considered as ancient dog breeds. Thus, the native population of Tibetan Terriers can be assigned to a limited group of the early canine foundation stock [3].

During the last two decades, a number of studies used different types of genetic markers to assess the genetic variation, heterozygosity and phylogenetic relationships between dog breeds [4]. In addition to the analysis of autosomal genomic regions, some studies also focused on the Y chromosome [5] and mitochondrial DNA (mtDNA) [6]. The first evidence on the genetic structure of dog populations and their phylogeny and on the genetic distances between breeds emerged from studies based on microsatellite markers (STR) [7–9], which separated several of the breeds with an ancient origin from the breeds with a modern European origin. The Tibetan Terrier breed was positioned into the group of ancient Asian breeds, close to the grey wolf. STR analysis can also be used to estimate population parameters and detect population events such as past bottlenecks [10].

With the availability of the complete canine genome sequence, a large number of single nucleotide polymorphisms (SNPs) was identified, which provided new opportunities for the genetic analysis of dog breeds [11]. Such analyses revealed the existence of long-range haplotypes across the entire canine genome and clarified the nature of the genetic diversity within and across breeds. An SNP-based analysis identified 51 regions in the dog genome that are associated with phenotypic variation of 57 traits [12]. An analysis of 509 dogs from 46 breeds revealed 44 genomic regions, which are associated with phenotypic traits that vary between breeds [13]. Recently, by investigating the genetic background of village dogs, Shannon et al. [14] reported the existence of a Central Asian domestication origin and identified geographic isolation, migration, and hybridization as important factors that shape the genetic diversity of village dog populations [14]. A genome-wide haplotype sharing analysis uncovered the geographic patterns of development and the independent origins of common traits in dogs [15]. Results based on genome-wide SNP genotypes are supported by those from whole-genome sequencing, which has recently become a powerful approach for association studies [4, 16] and for the study of traits related to adaptation [17, 18] and dog domestication [19, 20]. With the accumulation of genomic data, evolutionary and functional analyses on a finer scale can be carried out. Analysis of copy number variations (CNV) has also been used to detect genomic regions in the dog genome that are responsible for breed-specific phenotypes [21].

The positioning of Tibetan Terriers within the terrier group of dogs, which was based on a superficial phenotypic judgement of some cynologists in the early period of the formation of the breed, is often questioned today [2]. Neither historical nor genetic data support this decision. According to Ostrander [22], most of the terrier breeds fall within the “modern/hunting” cluster of dog breeds, which were all established from the same pool of ancestors in Europe in the nineteenth century. A few terrier breeds are found in the “mastiff” cluster, together with the Pomeranian and Labrador Retriever breeds. However, Tibetan Terriers cluster into the much older group of Asian and African dogs, along with the Pekingese breed [22]. Furthermore, some studies classify Tibetan Terriers into the group of ancient dog breeds [9], which is also known as the basal lineages [23]. A recent genome-wide analysis of Korean dogs [24] examined the genetic variation within Korean dog populations and their relationships with wolves and ancient and modern dog breeds. Among all the pairwise comparisons, the estimated F_ST_ value was highest (0.35) in the comparison between Tibetan Terriers and the Korean wolf and thus revealed their distant genetic relatedness.

The goal of our study was to provide an insight into the genomic structure and genetic history of Tibetan Terriers by using two unrelated and widely used types of genetic markers, high-density SNP microarray and microsatellite genotypes, to differentiate Tibetan Terrier lineages. In order to determine the genomic composition of the breed and its relationship to other breeds, we analysed the population structure of Tibetan Terriers and the potential admixture level between Tibetan Terriers (from both the western population of Tibetan Terriers and the native population in Tibet) and 33 other dog breeds, including the terrier and companion and toy dog groups, as well as the grey wolf, using publicly available datasets.

## Methods

### Sampling

For this study, we sampled buccal swabs from 64 Tibetan Terriers at dog shows or directly from breeders and owners: 24 western Tibetan Terriers (20 and 4 from the Lamleh and Luneville lineages, respectively), 22 native Tibetan Terriers, 8 TT-F1 (native × Lamleh), 6 TT-BC2 (F1 × Lamleh) and 4 TT-BC3 (TT-BC2 × Lamleh). The native Tibetan Terriers samples were collected at 22 locations in Tibet. Identification of purebred animals was based on specific morphological criteria and/or pedigree. DNA from buccal swabs was extracted using the standard protocol for the DNeasy Blood & Tissue kit (Qiagen, Germany). The quantity of DNA was estimated using NanoVue (GE Healthcare Life Sciences, USA).

### Genotypic data

We performed genotyping using a microsatellite set and an Illumina 170 k CanineHD BeadChip. The data obtained from genotyping were merged with the publicly available SNP array data for 5406 dogs belonging to 163 breeds downloaded from the Dryad repository [14]. The number of dogs per breed ranged from 1 to 732 (see Additional file 2: Table S1).

Quality control and merging of the SNP array data were done with the SNP and Variation Suite v8.7.0 (Golden Helix, Inc., Bozeman, MT, http://www.goldenhelix.com). First, we filtered the data obtained from the genotyping of 24 Tibetan Terriers by excluding the markers on the X chromosome (leaving 168,102 SNPs) and by removing the SNPs with a call rate lower than 95% and an Hardy–Weinberg equilibrium P-value less than 0.00001 (leaving 156,708 SNPs. Then, less than 10% of missing calls per sample were allowed, and 18 out of 24 Tibetan Terrier samples passed the quality control. After merging this dataset with the publicly available data, 143,170 SNPs that were common to both datasets, were available and distributed across all the autosomal chromosomes. The average marker density was one SNP every 15,404 kb. Based on publicly available data, we excluded the village dogs and mixed breed dogs, and finally selected 19 or less representative animals with the highest call rates for each breed. Tibetan Terriers were divided into five subpopulations (native, Lamleh, Luneville, F1 individuals between native and Lamleh Tibetan Terriers, and Tibetan Terriers that were extracted from the Dryad database [14].

To compare CanineHD BeadChip data with microsatellite data, we examined 18 microsatellite loci suggested by the International Society of Animal Genetics (ISAG): AHTK211, CXX279, REN169O18, INU055, REN54P11, INRA21, AHT121, FH2054, REN162C04, AHT137, REN169D01, AHTH260, AHTK253, INU005, AHTH171 and REN247M23, using the Canine Genotyping Panel 1.1 (Thermo Scientific, California) reagent kit. Genotyping was performed on the capillary Genetic Analyser ABI 3130xl. Microsatellite alleles were called using the GENEMAPPER v 4.0 software (ABI, Foster City, CA, USA) and manually checked.

### Neighbor-joining tree

We used various methods to provide a fine-scale assessment of the genetic structure of Tibetan Terriers. First, we computed pairwise Nei genetic distances between individuals belonging to 161 dog breeds (N = 1793) and grey wolf individuals (N = 14) using the ape R package [25]. A neighbor-joining tree was computed based on the distance matrix using the phyclust package for R [26].

### NeighborNet

Phylogenetic relationships between Tibetan Terriers and both dogs from 27 dog breeds (N = 349) and grey wolf individuals (N = 14) were analysed using the NeighborNet network based on Nei distances and the SplitsTree4 software [27]. We split Tibetan Terriers into two subpopulations, the native and western populations of Tibetan Terriers.

### Estimation of the migration rate using TreeMix

To infer the patterns of splits and mixtures in the populations of Tibetan Terriers and a subset of 28 dog breeds and the grey wolf, we used the Treemix algorithm [28] in which grey wolves (Gray_Wolf) were set as the rooting outgroup. First, we built a maximum likelihood tree of the populations with no migration events allowed. Then, we constructed a phylogenetic network for all selected populations, thereby increasing migration events sequentially up to 11 migrations. The residuals from the fit of the model to the data were visualized using the R script implemented in Treemix.

### Population structure and admixture analysis

To investigate the population structure inferred from SNP array and microsatellite genotyping data, we used the model-based clustering method STRUCTURE [29]. Visualisation of the results and estimation of the best K value according to Evanno [30] were performed using the web-based tool CLUMPAK [31].

SNP array data were used to investigate the population structure of two datasets: (1) 366 dogs from 29 breeds and 14 grey wolves (by merging genotyped samples from this study and publicly available samples), and (2) a subset that included Tibetan Terrier samples only. We pruned the CanineHD BeadChip data from the 143,170 SNPs by setting a window size of 100, a step size of 20 and a pairwise r^2^ threshold of 0.1, which left data for 22,175 SNPs. A model that assumes admixture and correlated allele frequencies was used for all STRUCTURE runs, with a burn-in of 10^6^ iterations followed by 10^5^ MCMC iterations. Runs were repeated 10 times for each K value, which ranged from 1 to 20 for the larger dataset (29 dog breeds and grey wolf) and from 1 to 6 for the subset of Tibetan Terrier samples.

The STRUCTURE program was used to estimate the number of genetic populations/lineages in Tibetan Terriers based on allele frequencies of microsatellites with a burn-in of 10^6^ iterations followed by 10^5^ MCMC iterations. Runs were repeated 10 times for each K value ranging from 1 to 6. All Tibetan Terrier samples included in the SNP array analysis were present in the microsatellite dataset. However, the microsatellite dataset consisted of additional samples that were not genotyped on the SNP array.

### Runs of homozygosity

Runs of homozygosity (ROH) were identified for each individual separately using the SNP and Variation Suite v8.8.3 (Golden Helix, Inc., Bozeman, MT, http://www.goldenhelix.com). ROH were defined as runs of 25 or more homozygous SNPs that had a minimum run length of 500 kb and in which no heterozygous SNPs, and no more than five missing SNPs were allowed. Similar to other studies [32], ROH were summarized in three length categories: short ranging from 0.5 to 2.5 Mb; medium ranging from 2.5 to 5.0 Mb; and long i.e. more than 5 Mb. Genomic inbreeding coefficients (F_ROH_) were calculated as in [33] and were defined as the percentage of the autosomal genome present in a ROH. The length of the autosomal genome was set to 2,392,715,236 bp.

## Results

### Assessment of population structure using tree-based methods

**Fig. 1:**
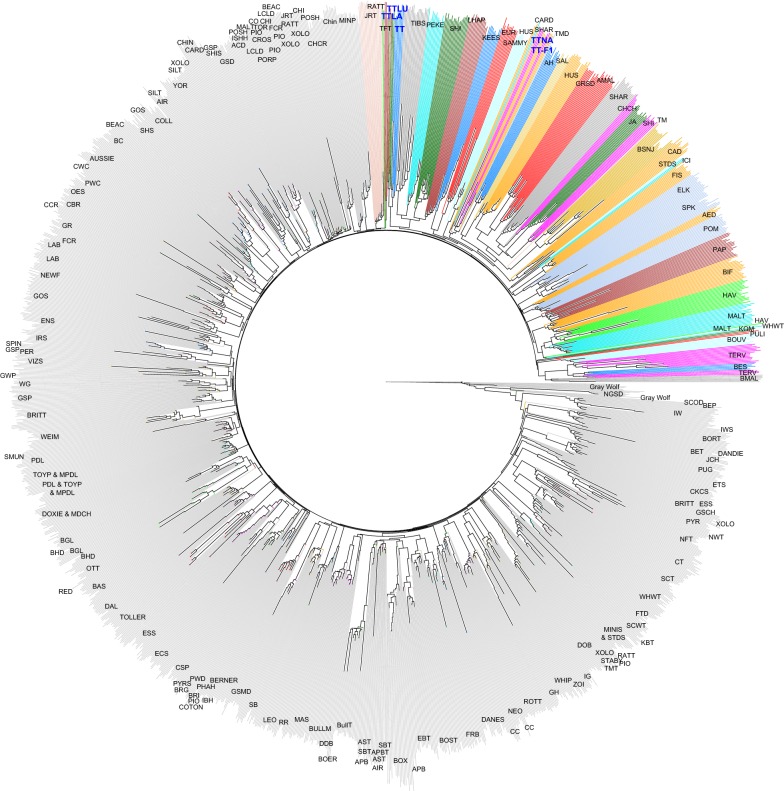
Relationship of Tibetan Terriers with other dog breeds. Neighbor-joining tree based on SNP chip data for 161 dog breeds revealing a compact cluster of ancient dog breeds including native Tibetan Terrier and F1 crosses with its western lineages. The western lineages are part of the Tibetan Companion Dog cluster

Based on CanineHD Illumina SNP chip data, we analysed the relationships between Tibetan Terriers and selected dog breeds by constructing a neighbour-joining tree from raw genetic distances. This analysis revealed several main characteristics of the population structure and showed that dogs from the same breed almost invariably built compact clusters. This reflects the fact that modern breeds mainly represent closed gene pools that have experienced major population bottlenecks during their formation. We detected relatively little structuring within the internal branches that distinguish the breeds. This is consistent with the suggestion that all modern dog breeds arose from a common ancestral population during a relatively short period, and that only a small proportion of the genetic variation divides dog breeds into subgroups. Tibetan Terriers formed two clusters: cluster (1) includes the native population and its F1 cross with western Tibetan Terriers, which are grouped together with the more ancient dog breeds such as Afghan Hound, Samoyed, Sloughi and Siberian Husky, and cluster (2) includes the western lineages (Lamleh and Luneville), which are part of the Tibetan Companion Dog cluster, including Tibetan Spaniel, Pekingese, Shih Tzu, and Lhasa Apso, and showed some relatedness with the group of Terrier breeds. This suggests that some sporadic contacts may have occurred between the two western lineages of Tibetan Terriers and some Terrier breeds during the last century. The internal branches leading to the populations of Boxer and grey wolf are longer than those leading to other breeds. The long Boxer branch may be explained by the fact that a large proportion of the SNPs on the Canine HD array was selected by comparing Boxers with other breeds, which implies that the dataset is enriched for SNPs that allow the differentiation of Boxers from other breeds. The longer grey wolf branch probably reflects their more distant relatedness to dogs (Fig. 1).

Some breeds show a clear tendency to build separate clusters, such as Retrievers, Spaniels, Setters, and Terriers. However, the length of the internal branches leading to these clusters represents only a small portion of the average total length of the branches in these clusters, which indicates that major bottlenecks occurred during their formation, although in general the detailed analysis of these data supports a common historical origin of the related breeds. The tree is consistent with previous studies and supports the accuracy and reliability of the genotypes obtained by using HD micro array analysis. Although the long Boxer branch most likely reflects the SNP ascertainment bias of the array, this tree indicates a high level of polymorphism, both within and between breeds. This supports the expectation that the SNP ascertainment bias does not hinder the detection of genetic variation within and between breeds.

### Phylogenetic analysis—NeighbourNet

**Fig. 2:**
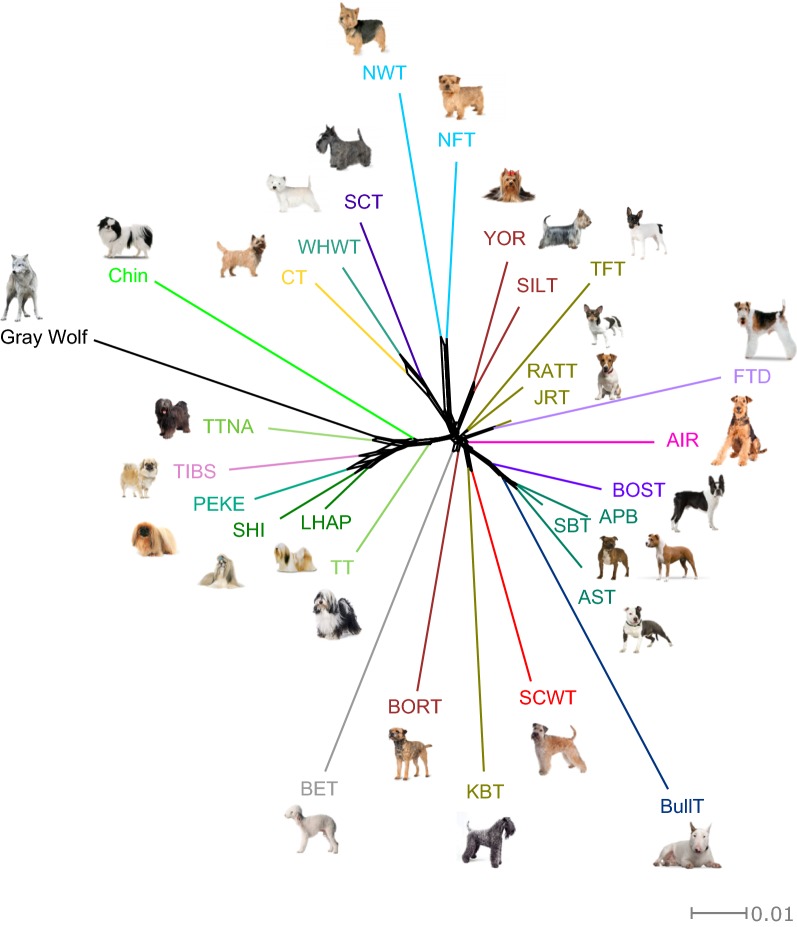
NeighborNet network relating 27 dog breeds and Grey Wolf. NeighborNet network, based on Nei’s genetic distances, reveals the relationships between 27 dog breeds and Grey Wolf. Reticulations on the graph indicate past hybridization events between populations. Breed names are abbreviated as follows: Airedale Terrier (AIR), American Pit Bull Terrier (APB), American Staffordshire Terrier (AST), Bedlington Terrier (BET), Border Terrier (BORT), Boston Terrier (BOST), Bull Terrier (BullT), Cairn Terrier (CT), Fox Terrier Wire (FTD), Jack Russell Terrier (JRT), Japanese Chin (Chin), Kerry Blue Terrier (KBT), Lhasa Apso (LHAP), Norfolk Terrier (NFT), Norwich Terrier (NWT), Pekingese (PEKE), Rat Terrier (RATT), Scottish Terrier (SCT), Shih Tzu (SHI), Silky Terrier (SILT), Soft Coated Wheaten Terrier (SCWT), Staffordshire Bull Terrier (SBT), Tibetan Spaniel (TIBS), Native Tibetan Terrier (TTNA), Tibetan Terrier (TT), Toy Fox Terrier (TFT), West Highland White Terrier (WHWT), Yorkshire Terrier (YOR)

To provide additional insight into the origin and evolutionary history of Tibetan dog breeds, we constructed a NeighborNet network based on Nei’s genetic distances (Fig. 2). Breeds belonging to the same group of FCI breed (Terriers) clustered together. All Tibetan Terrier populations were also grouped together and the native population of Tibetan Terriers branched closer to the grey wolf than any other breed. The Tibetan Spaniel, Pekingese, Shih Tzu, and Lhasa Apso branches were the populations of native and western Tibetan Terriers, while Japanese Chin was on its own branch. Sets of parallel edges on the NeighborNet network observed between the population of native Tibetan Terriers and other Tibetan Toy breeds (Tibetan Spaniel, Pekingese, Shih Tzu, and Lhasa Apso) indicate hybridization events between them. The other Terrier breeds formed a separate group on the other side of the network.

### Treemix

Using the Treemix software, we modelled both population splits and gene flow between a subset of 29 dog breeds and the grey wolf. As explained in “Methods” section, Tibetan Terriers were divided into five subgroups: native Tibetan Terriers, the two western lineages Lamleh and Luneville of Tibetan Terriers, F1 individuals between the western lineages and native population, and Tibetan Terrier samples that were extracted from the Dryad database (datadryad.org) [14].

**Fig. 3:**
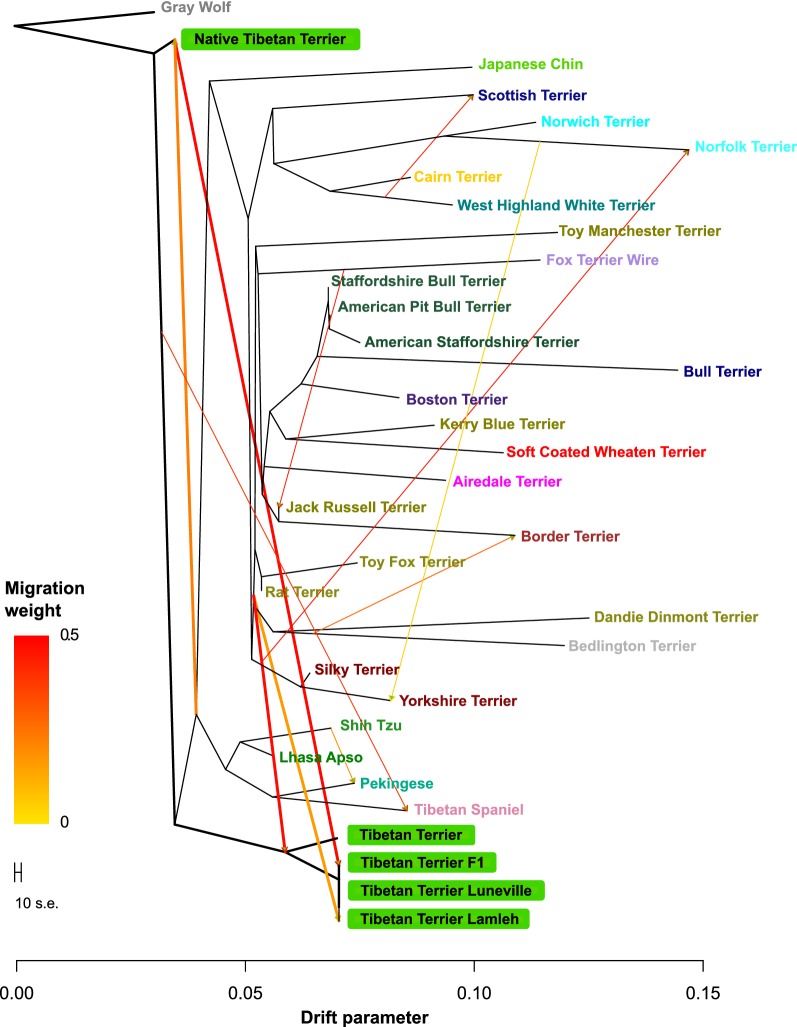
Migrations within the Asian toy dog and Terrier breeds. Maximum likelihood tree showing the most important migrations within the Asian toy dog- (Tibetan Terrier, Japanese Chin, Shih Tzu, Lhasa Apso, Pekingese, and Tibetan Spaniel) and Terrier breeds

Our results support the hypothesis that the native Tibetan Terriers are closer to the grey wolf than to any other dog breed analysed here. We detected significant gene flow from the native population of Tibetan Terriers to both Lamleh and Luneville lineages, the F1 individuals and to the Tibetan Terriers from the publicly available genotypes in the Dryad database [14] (Fig. 3). We also detected some migration of genes from the Terrier’s ancestor but only to the breeds positioned at the beginning of the European lineage branch. In addition, we observed a few less significant migration events to the Lamleh lineage only, which might be due to breeders assuming that the Tibetan Terrier belongs to the Terrier group and thus to a few sporadic crossings between Tibetan Terriers and Terriers during the last century. In addition, the Lamleh and Luneville lineages, the F1 individuals, and the Tibetan Terriers retrieved from Dryad database clustered together with other Tibetan dog breeds, rather than with Terrier breeds.

### STRUCTURE analysis

**Fig. 4:**
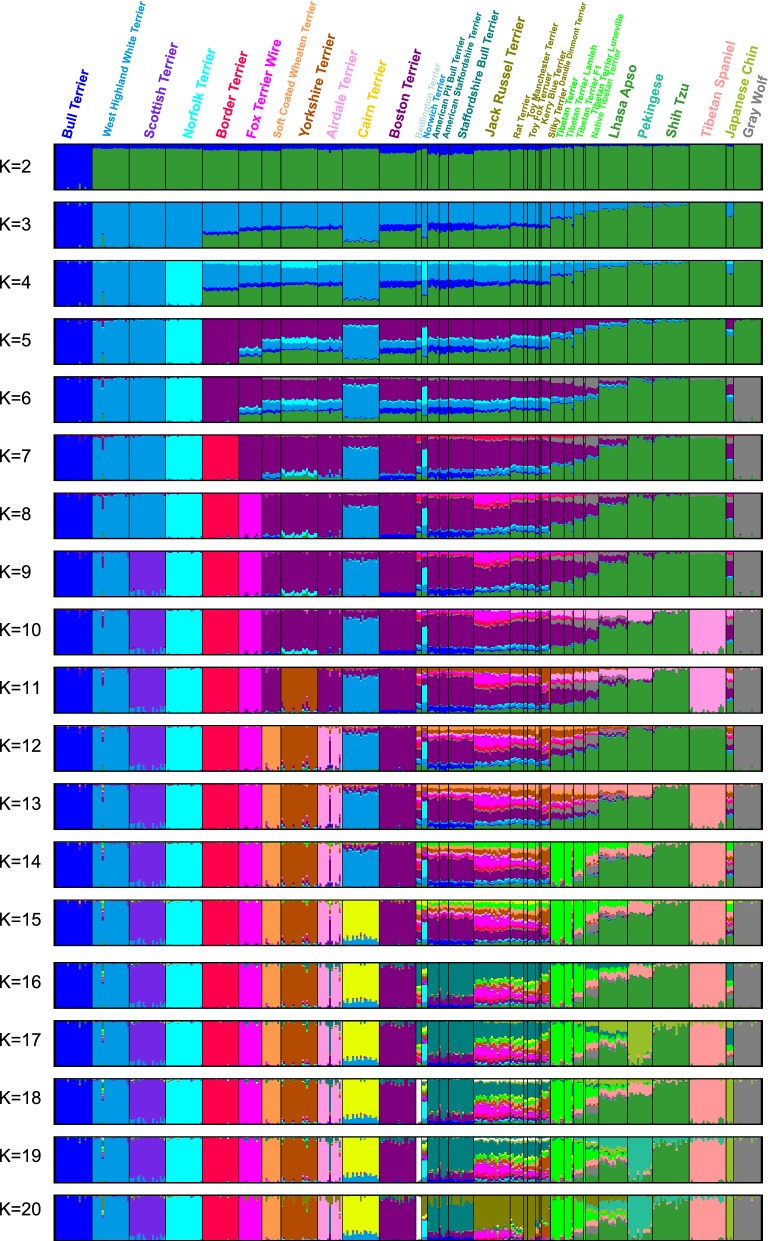
Genetic structure of Terriers and companion dogs. Bayesian clustering on a complete CanineHD BeadChip dataset of 29 dog breed populations and Grey Wolf (N = 366) performed with STRUCTURE and visualized using the CLUMPAK software

The STRUCTURE plots for K values from 2 to 20 are in Fig. 4. In each plot, each cluster is indicated by a different colour and each individual is shown as a vertical bar divided into at most K coloured segments with heights being proportional to genotype memberships. Western Tibetan Terriers show more uniformity than native Tibetan Terriers, which display other colours that represent genetic elements, also present in other populations (Shih Tzu, Grey Wolf, Tibetan Spaniel, Japanese Chin, western population of Tibetan Terriers, Pekingese and some Terriers).

**Fig. 5:**
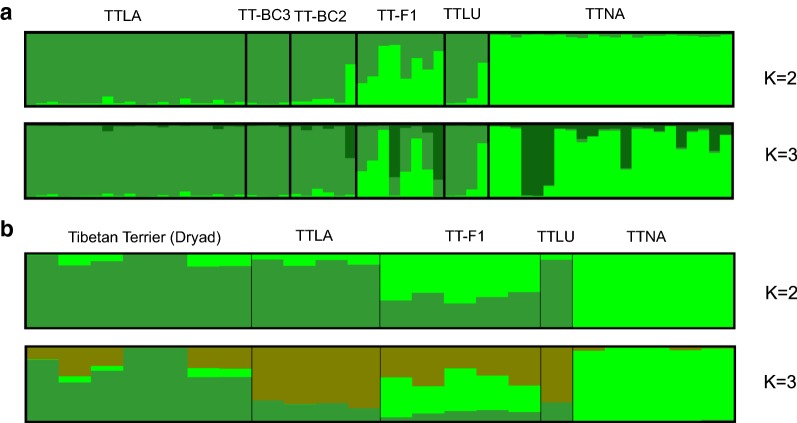
STRUCTURE analysis of Tibetan Terrier subpopulations. STRUCTURE analysis of microsatellite (**a**) and SNP chip data (**b**) from different Tibetan Terrier subpopulations. Individuals were assigned to clusters at different K values. Breed abbreviations: TTLA: Tibetan Terrier, Lamleh lineage; TTLU: Tibetan Terrier, Luneville lineage; TTNA: Tibetan Terrier, native population; TT-F1. TT-BC2, TT-BC3: F1 and two back cross generations from the crossing of TTNA with the western TT

In order to compare the results of the analysis of population structure of Tibetan Terriers, obtained with different types of markers, we performed a STRUCTURE analysis based on microsatellite (Fig. 5a) and SNP chip data (Fig. 5b). This analysis showed that, within the population of Tibetan Terriers, individuals are genetically very similar. For the native population of Tibetan Terriers, there was a 95% contribution of one genetic cluster and a 5% contribution of the other genetic cluster, whereas the opposite was observed for the Tibetan Terrier Luneville lineage. The TT-F1 cross showed contributions between 20 and 80% of each subpopulation, TT-BC2 showed a 90% contribution of the second subpopulation, and TT-BC3 had almost 100% of the genotype that prevails in the western subpopulation.

The best K according to Evanno [30] was used to determine the most likely value of K i.e. 3. Therefore, although our populations of Tibetan Terriers differ, they actually consist of three genetic clusters: the native Tibetan and western cluster, which shows some within-cluster structure. The genetic basis of the native population of Tibetan Terriers is wider than that of the western population of Tibetan Terriers, however, only the superficial evaluation of the phenotype does not clearly support this fact. The existence of a broader genetic pool in the native population of Tibetan Terriers is highly relevant for possible prevention measures against increases in homozygosity and risk for genetic diseases.

STRUCTURE analysis of the six Tibetan Terrier subpopulations based on microsatellite data and SNP chip data resulted in similar substructures in all subpopulations. Due to the extreme bottleneck that led to the western population of Tibetan Terriers, it can be clearly differentiated from the native population of Tibetan Terriers. Both ancestral gene pools are present in the F1, F2 and F3 generations and, at K = 3, substructuring in the native population becomes apparent.

### Estimation of inbreeding coefficients based on ROH analysis

**Fig. 6:**
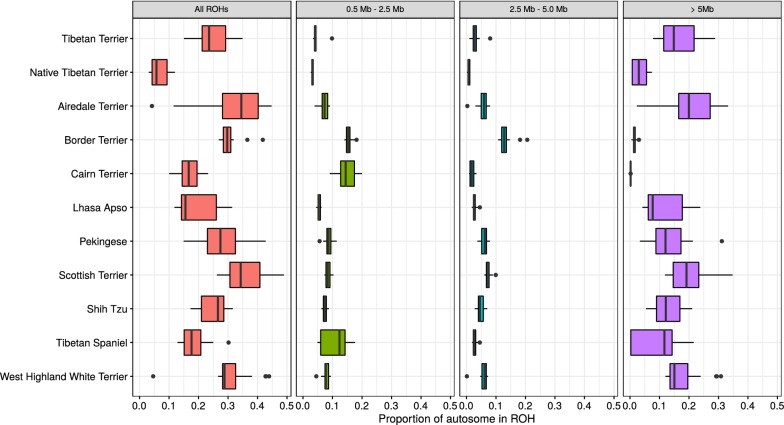
Proportion of the autosomal genome in ROH. Proportion of the autosomal genome in ROH for 11 dog populations; considering all ROH (first panel) and ROH summarized in three length categories: short = between 0.5 and 2.5 Mb; medium = between 2.5 and 5.0 Mb; and long = more than 5 Mb

Estimated inbreeding coefficients based on ROH analysis (F_ROH_) were very low for the native population of Tibetan Terriers compared to those of several other breeds (Fig. 6). Compared to the western population of Tibetan Terriers, the native population is characterized by a small number of long ROH and by a low proportion of the genome covered by ROH i.e. 0.07 instead of 0.24 in the western population. Assessment of the distribution of ROH lengths among populations gives an indication of the timing of diversity loss, and in our study, we found a larger fraction of the genome covered by long ROH in the western population of Tibetan Terriers, which indicates recent inbreeding. Thus, based on this ROH analysis, we show that the native population of Tibetan Terriers belongs to the dog populations with the lowest level of inbreeding, which increases its value as genetic resource.

## Discussion

### Analysis of the population structure and genetic relationships of Tibetan Terriers with other breeds

Tibetan Terrier is one of the very few dog breeds, for which the native ancestral population is available in Tibet that represents the original gene pool from which originates the modern western population of Tibetan Terriers. The western population experienced a major bottleneck at the beginning of its formation, which resulted in a relatively rapid increase in inbreeding and in several hereditary health problems. Moreover, when the western population of Tibetan Terriers was established as a breed, based on its phenotypic features, it was placed within the group of Terriers. According to population genetics studies, most terriers are included in the “modern/hunting” cluster of dog breeds that were developed from the same pool of ancestors in Europe in the nineteenth century, whereas the Tibetan Terrier belongs to the older group of Asian and African dogs, along with the Pekingese breed [22]. Several studies classified the Tibetan Terrier as an ancient breed due to its high level of divergence compared to that of other dog breeds. It is believed that the Tibetan Terrier breed originated about 500 years ago and that it was highly associated with the original domestication of dogs [23]. Thus, Tibetan Terrier can be considered as one of the basal lineage breeds of domestic dogs and as a live prototype of ancestral dogs. The recent study by [24], which focuses on the origin, diversity and population structure of Korean dogs, includes Tibetan Terriers and supports the genetic relatedness between the Korean wolf and Tibetan Terrier. This study reports a neighbour-joining tree with the coyote at the root of the tree and shows that Tibetan Terrier belongs to the same branch as Afghan Hound, Basenji, Lhasa Apso, and Shi Tzu, which supports the findings of [9].

Our results clearly show migratory events between the native and western populations of Tibetan Terriers. There are also visible exchanges of genetic material from Terrier ancestors to the western population of Tibetan Terriers, which impacted only the beginning of the European Tibetan Terrier branch. This might be because, in the past, the name “Terrier” was mistakenly used, which led the breeders of Tibetan Terriers to cross Tibetan Terriers with some other Terrier ancestors. In addition, the fact that Tibetan Terriers are placed closer to other Tibetan dog breeds than to other Terriers confirms that the dog today known as Tibetan Terrier, is not a terrier at all.

Our results demonstrate that the Tibetan Terrier and its lineages build a cluster, which is close to other ancient breeds of Asian origin and the grey wolf. This confirms the ancient character of the Tibetan Terrier and its positioning within the cluster of ancient breeds, i.e. close to the domestication event. Today’s population of Tibetan Terriers is clearly split into two populations: the native and western populations of Tibetan Terriers. The native populations of Tibetan Terriers represents the main gene resource for the western population of Tibetan Terriers, which has been separated from the original population for almost 100 years as a consequence of a major bottleneck and genetic drift. Although less obvious, a contribution of Terriers to the western population Tibetan Terriers can be assumed. According to the STRUCTURE analysis, Tibetan Terrier clearly belongs to the Asian ancient cluster, which also contains a grey wolf gene pool. The more K increases, the clearer is the influence of Terriers on the western population of Tibetan Terriers.

The basic structure of the Tibetan Terrier populations is supported by both SNP and STR data. The intermediate position of the F1 individuals and the position of two back cross generations closer to the western lineages clearly show the effect of genetic drift in the Lamleh Tibetan Terrier lineage and that some minor alleles from the native population of Tibetan Terriers are represented in the Lamleh Tibetan Terrier lineage. The larger effective size of the native population of Tibetan Terriers and the breeding practices supporting high levels of admixture resulted in a relatively low inbreeding level compared to the western population of Tibetan Terriers, which has a much higher inbreeding level due to its small gene pool and the western breeding practices. These characteristics are also reflected by the proportion of the genome covered by ROH, i.e. the fraction of the genome containing homozygous regions is much smaller for the native population of Tibetan Terriers than for the western lineages. The genetic diversity of the native population of Tibetan Terriers compared to its western derivatives is higher, which is indicated by the very low proportion of the genome in ROH for the native population of Tibetan Terriers (Fig. 6).

Our findings show that the level of genetic diversity differs between the original native population of Tibetan Terriers and its western derivatives. The original population represents a natural gene pool resource, which could be used to reduce the level of inbreeding in the western population of Tibetan Terriers and represents an important genetic resource to preserve the original genetic diversity and to improve that of the western population of Tibetan Terriers.

## Conclusions

Our results reveal the considerable difference in genetic diversity between the original native population of Tibetan Terrier and its western population. The genetic pool of the western population of Tibetan Terriers represents only a tiny part of that of the original population. Because of its isolation from the original population and the genetic drift operating in its population with a small effective size, it can be clearly separated from the original population of Tibetan Terriers. This original population of Tibetan Terriers with its very low proportion of the genome included in long runs of homozygosity represents a unique example of existing contemporary ancestral and modern western populations, the latter being managed according to cynological practices during the last century. The native population of Tibetan Terriers represents also a backup population for the western population of Tibetan Terriers and can be used to significantly reduce the level of inbreeding of the latter. Thus, it is an important genetic resource for preserving the original gene pool and for improving the genetic diversity of the western population of Tibetan Terriers.

## Supplementary information

**Additional file 1: Figure S1.** Tibetan Terrier lineages. The Lamleh lineage can be traced back to 1922 when Agnes Grieg acquired the first couple of Tibetan Terriers (Bunti and Rajah) and brought them to England in 1930. The Kennel Club in England recognized the Tibetan Terrier as its own breed in 1937. The founders of the Luneville lineage were Dusky, a stray dog, found by John Downey in Liverpool in 1953 and registered by the English Kennel Club as a Tibetan Terrier Troyan Kynos and the bitch Princess Aureus. Images from: http://www.tibetan-terrier.org.

**Additional file 2: Table S1.** Breeds and number of dogs included in the analyses. The breeds and number of dogs per breed, used in this study are listed.

## Data Availability

The datasets generated and analysed during the current study will be available in the Dryad repository.
