# Supplementary material for: Population structure and genetic history of Tibetan Terriers
Source: Genet Sel Evol. 2019 Dec 27;51:79. doi: 10.1186/s12711-019-0520-4 (PMC6935067; doi:10.1186/s12711-019-0520-4)
Supplement: Supplementary file 1 — Additional file 1: Figure S1. Tibetan Terrier lineages. The Lamleh lineage can be traced back to 1922 when Agnes Grieg acquired the first couple of Tibetan Terriers (Bunti and Rajah) and brought them to England in 1930. The Kennel Club in England recognized the Tibetan Terrier as its own breed in 1937. The founders of the Luneville lineage were Dusky, a stray dog, found by John Downey in Liverpool in 1953 and registered by the English Kennel Club as a Tibetan Terrier Troyan Kynos and the bitch Princess Aureus. Images from: http://www.tibetan-terrier.org. [file 12711_2019_520_MOESM1_ESM.pdf]

The founders of Lamleh lineage

Rajah

Bunti

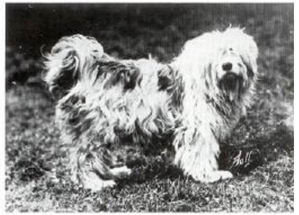

The founders of Luneville lineage

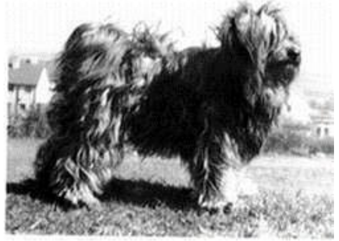

Dusky Troyan Kynos, a stray dog found in Liverpool in 1953

Princess Aureus

Lamleh — 1930

Luneville — 1953
